# Supplementary material for: Characterization of Hantavirus N Protein Intracellular Dynamics and Localization
Source: Viruses. 2022 Feb 23;14(3):457. doi: 10.3390/v14030457 (PMC8954124; doi:10.3390/v14030457)
Supplement: Supplementary file 1 [file viruses-14-00457-s001.zip › viruses-1562753-supplementary/viruses-1562753-supplementary.pdf]

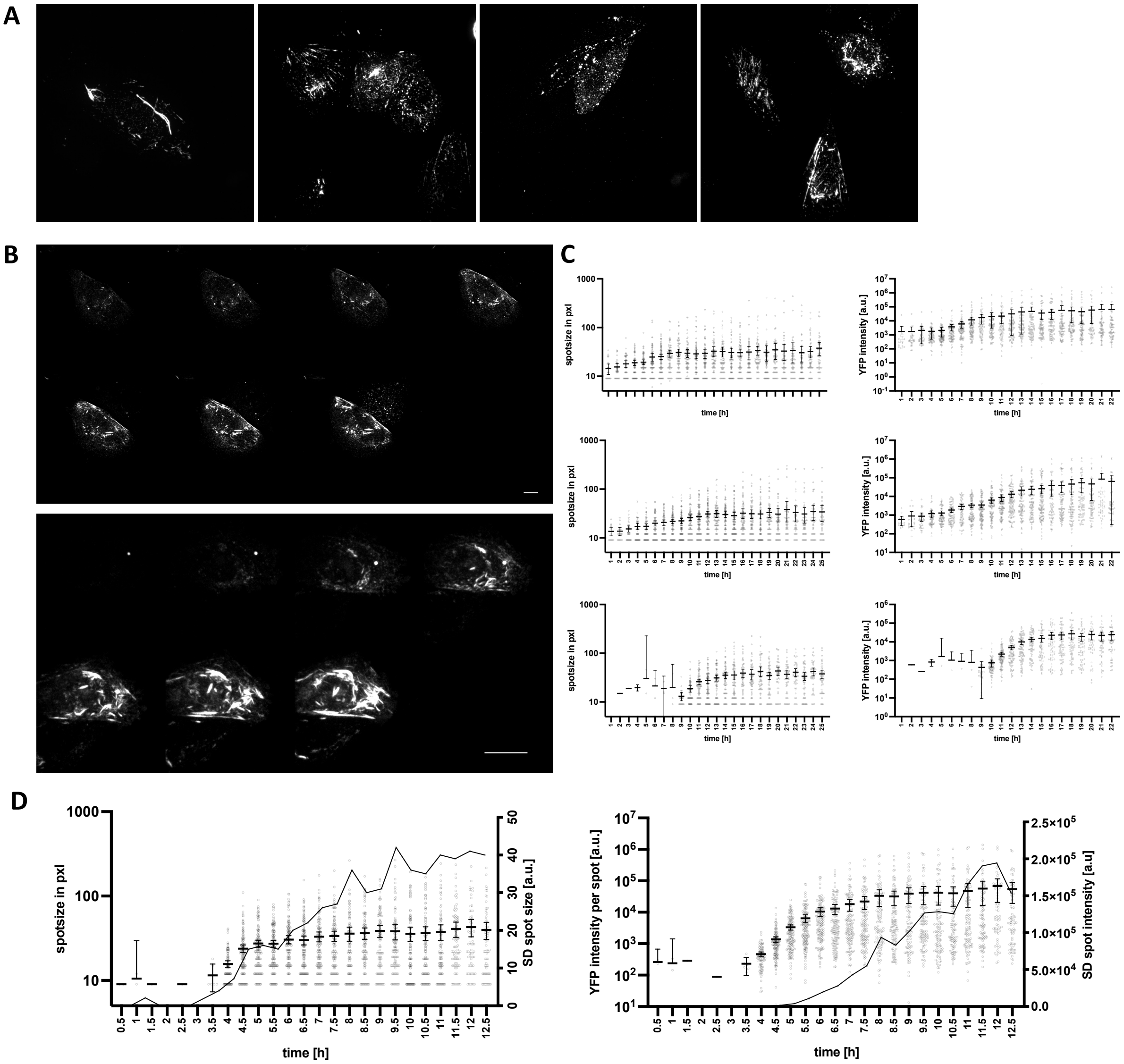

Fig S1: N protein heterogeneity and dynamics of YFP-N expression patterns. (A) Non-tagged N protein construct were transfected into CHO-K1 cells and subjected to immunofluorescence staining using anti-N protein antibodies and then to spinning disk microscopy. **(B)** CHO-K1 cells transfected with YFP-N and imaged by spinning disk microscopy. The Panel shows additional examples for manuscript figure 1. Individual cells were repeatedly imaged for 12.5 h (30 minutes intervals) post transfection and displayed as tiled-images. Two representative cells are shown on the left. All micrographs show maximum intensity projections of z-stacks. Scale bars: 10 µm. **(C)** Protein aggregates were identified by automated image analysis using the imageJ plugin ComDet. Spot size and fluorescence intensity are plotted over time (right panel). Plots show individual detected spots as grey dots. Bars show mean with 95% confidence interval. **(D)** Spot size and YFP intensity per spot as shown in Figure 1. Solid lines indicate SD.

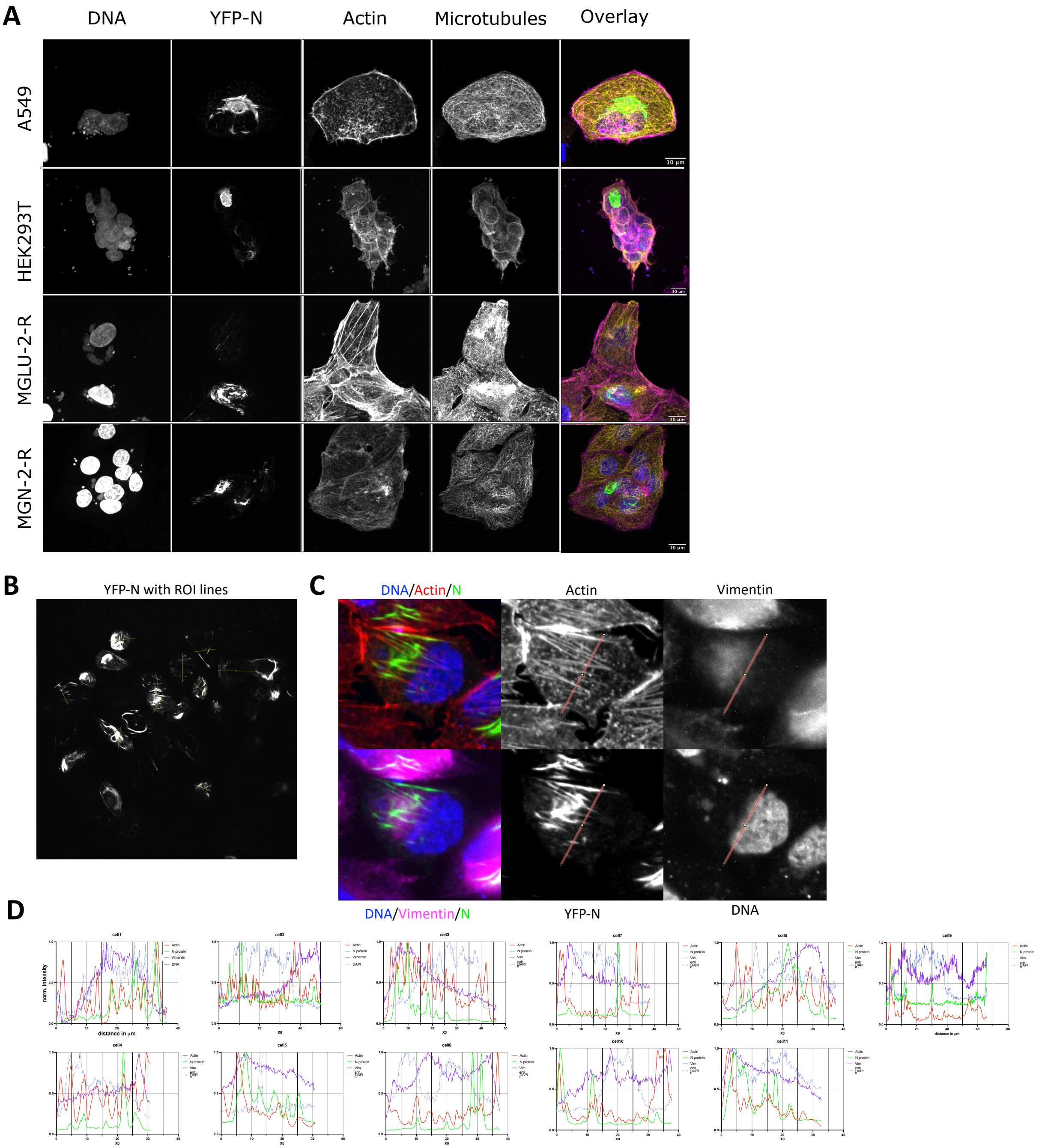

Fig S2: YFP-N co-localizes with cellular actin and vimentin. Supplementary data and additional examples for Figure 2. **(A)** The bank vole cell lines MGLU-2-R (lung derived), MGN-2-R (kidney derived), and the human epithelial cell lines A549 and Hek293T were transfected with YFP-N and stained for actin using Rhodamine-Phalloidin as well as microtubules and vimentin by immunofluorescence staining. Large aggregates and spike-like structures with actin-colocalization were found, irrespective of the cell type. **(B)** CHO-K1 cells were transfected with YFP-N and stained for actin using Rhodamine-Phalloidin and vimentin by immunofluorescence. Overview image shows multple cells with regions of interests (ROIs, in yellow) for line plots. **(C)** Representative cell as shown in Figure 2 with additional overlays. **(D)**

Line-plot analysis of multiple individual cells after normalization. Line plots were utilized for correlation analysis as shown in Figure 2B. Scale bars: 10 µm.

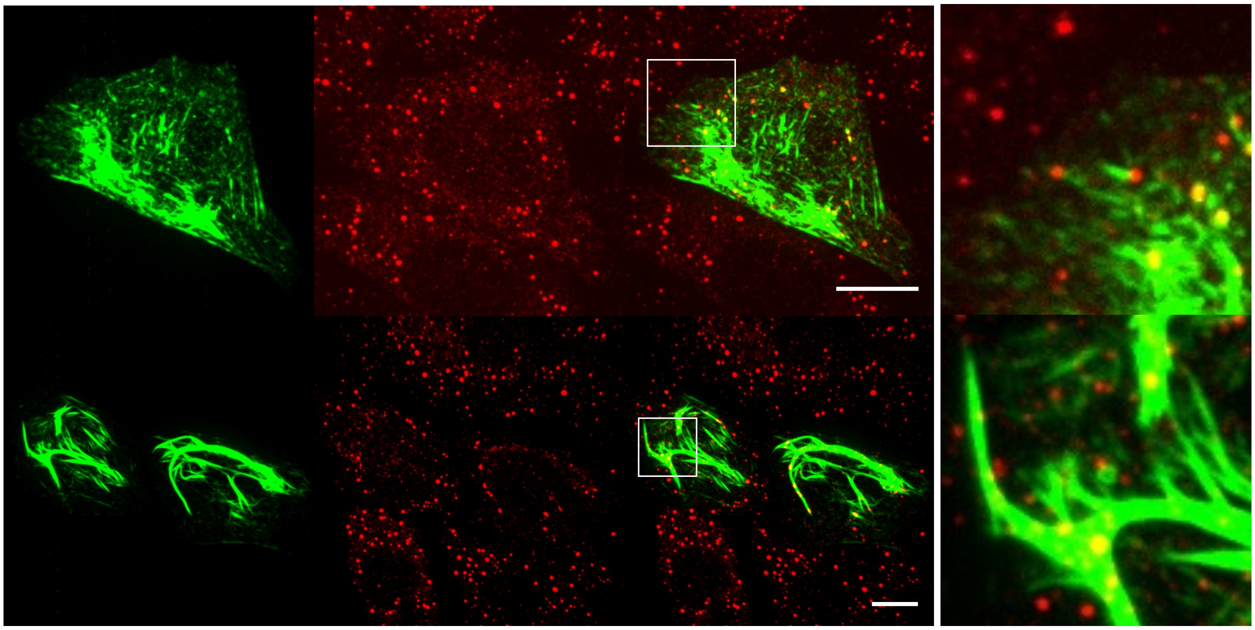

Fig S3: YFP-N co-localizes with P-bodies. CHO-K1 cells were transfected with YFP-N and stained for P-bodies by immunofluorescence. The boxed areas are shown magnified on the left. All images show maximum intensity projections of z-stacks. Scale bars: 10 µm.

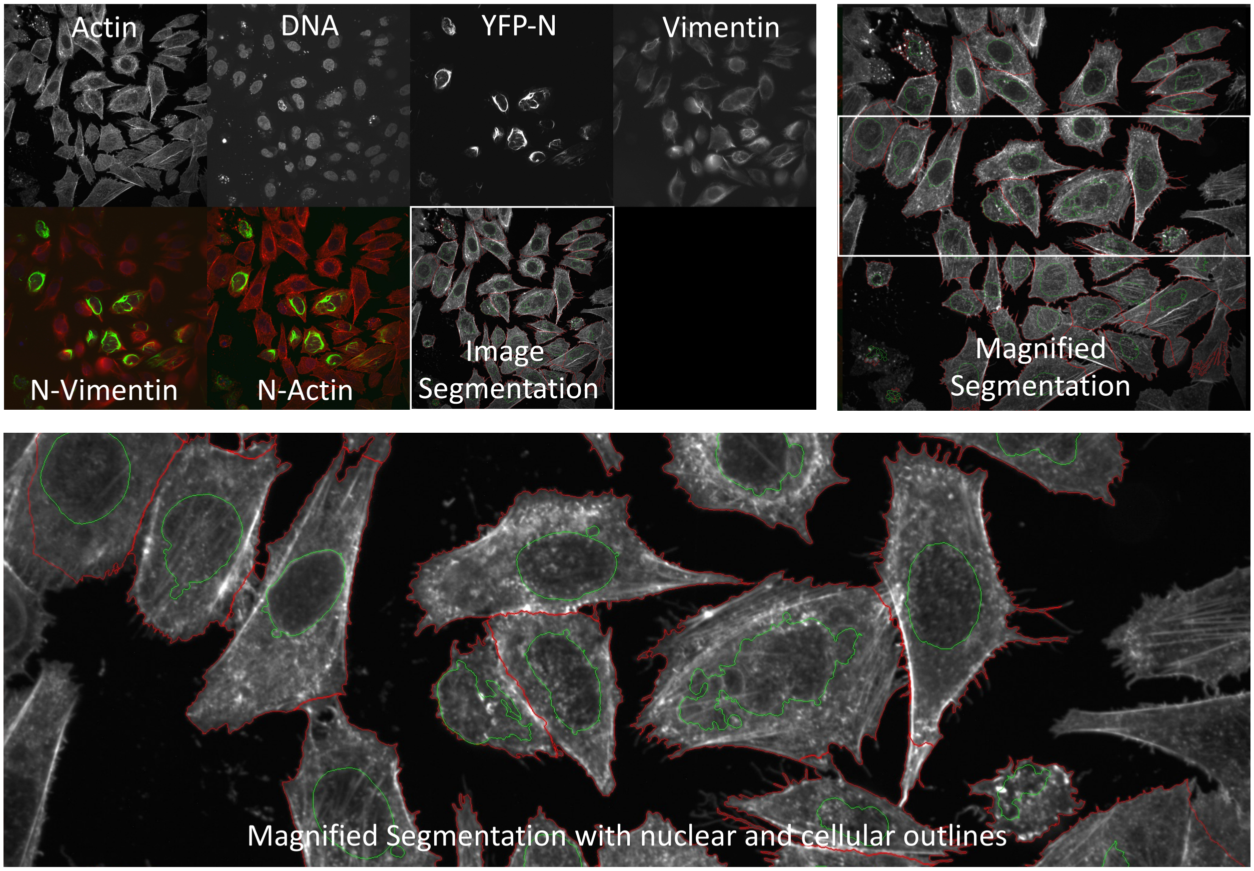

Fig S4: Quantitative image analysis and segmentation. Representative cell profiler output showing image segmentation. Identified nuclei (green) and cells (red) are overlayed on actin stainings.

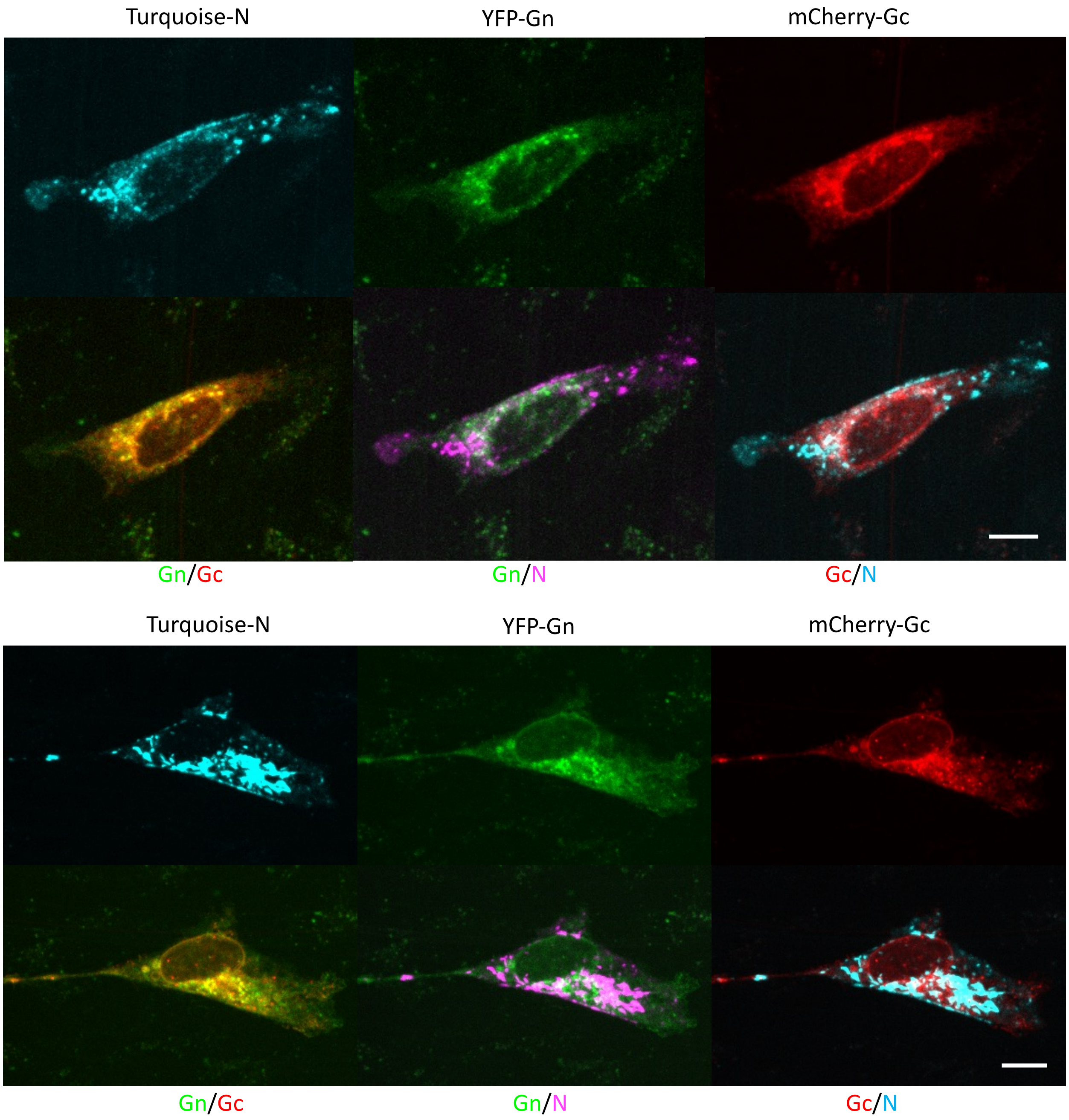

Fig S5: Turquoise-N co-localizes with fluorescently tagged Gc and Gn. CHO-K1 cells were transfected with Turquoise-N, YFP-Gn and mCherry-Gc. Additional example for Figure 5. Scale bars: 10 µm.
