# Supplementary material for: Characterization of Hantavirus N Protein Intracellular Dynamics and Localization
Source: Viruses. 2022 Feb 23;14(3):457. doi: 10.3390/v14030457 (PMC8954124; doi:10.3390/v14030457)

Plasmid Map

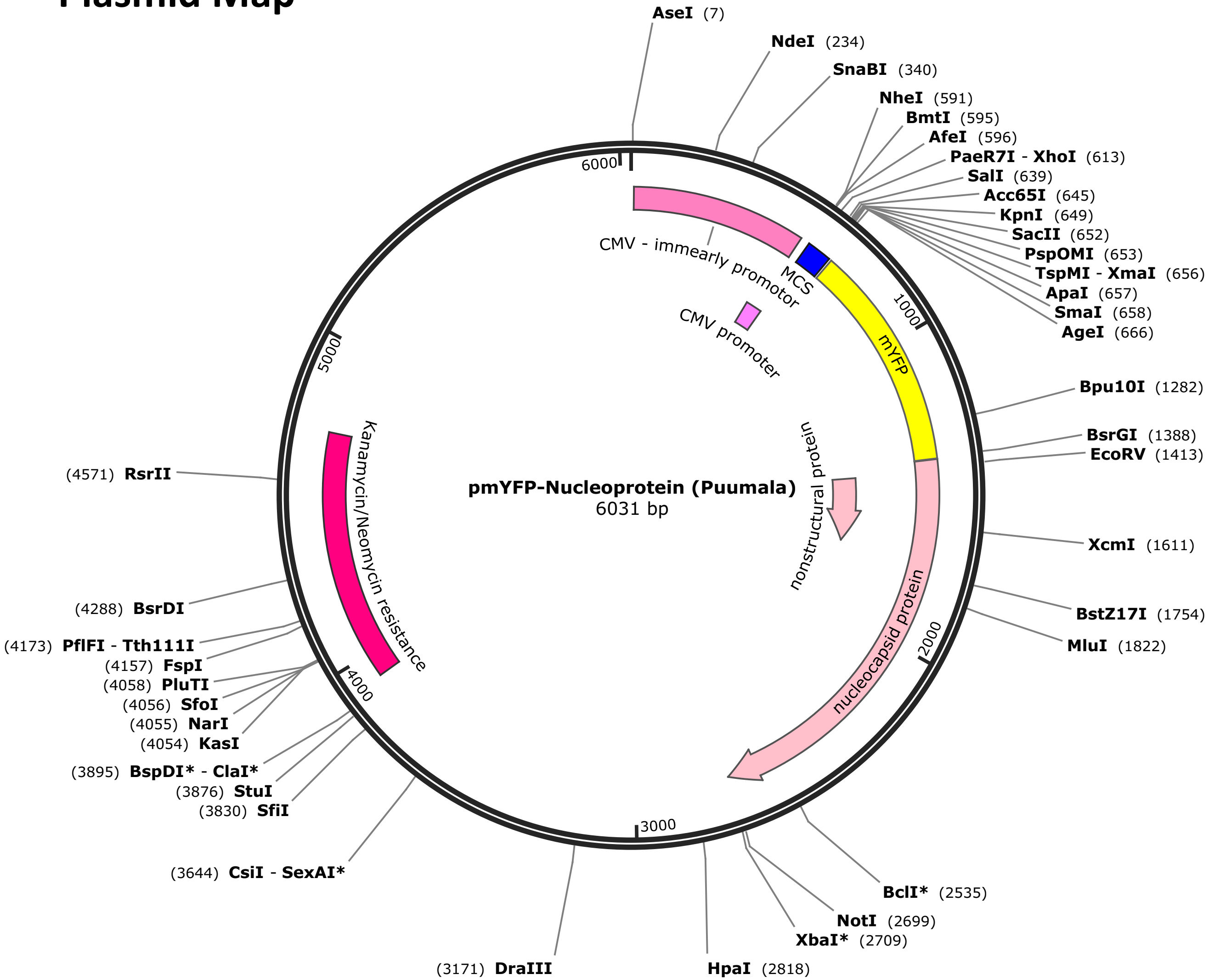

Primary Sequence.

MVSKGEELFTGVVPILVELDGDVNGHKFSVSGEGEGDATYGKLTLKFICTTGKLPVPWPTLVTTFGYGLQCFA
RYPDHMKQHDFFKSAMPEGYVQERTIFFKDDGNYKTRAEVKFEGDTLVNRIELKGIDFKEDGNILGHKLEYN
YNSHNVYIMADKQKNGIKVNFKIRHNIEDGSVQLADHYQQNTPIGDGPVLLPDNHYLSYQSKLSKDPNEKRD
HMVLLEFVTAAGITLGMDELYKMSDLTDIQEDITRHEQQLIVARQKLKDAERAVEVDPDDVNKNTLQARQQ
TVSALEDKLADYKRRMADAVSRKKMDTKPTDPTGIEPDDHLKERSSLRYGNVLDVNAIDIEEPSGQTADWYT
IGVYVIGFTLPIILKALYMLSTRGRQTVKENKGTRIRFKDDTSFEDINGIRRPKHLYVSMPTAQSTMKAEELTPG
RFRTIVCGLFPTQIQVRNIMSPVMGVIGFSFFVKDWSERIREFMEKECPFIKPEVKPGTPAQEIEMLKRNKIYF
MQRQDVLDKNHVADIDKLIDYAASGDPTSPDNIDSPNAPWVFACAPDRCPPTCIYVAGMAELGAFFSILQD
MRNTIMASKTVGTAEEKLKKKSSFYQSYLRRTQSMGIQLDQRIILLFMLEWGKEMVDHFHLGDDMDPELRG
LAQALIDQKVKEISNQEPLKI*

mYFPSpacerN protein

Construct Schematics

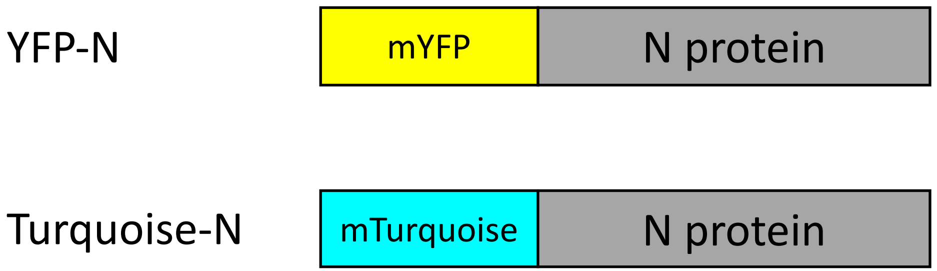

Supplement: Supplementary file 1 [file viruses-14-00457-s001.zip › viruses-1562753-supplementary/viruses-1562753-supplementary.pdf]
